# Descriptive study of patients admitted to acute psychiatry care

**DOI:** 10.1192/j.eurpsy.2025.1624

**Published:** 2025-08-26

**Authors:** P. S. Pires, C. Cunha, R. Cabral, F. Cunha, I. Santos, A. P. Costa

**Affiliations:** 1 DPSM, ULS Viseu Dão-Lafões, Viseu, Portugal

## Abstract

**Introduction:**

As a first-year resident doctor in specialized psychiatry training in Portugal, I have begun my inpatient internship, which is the longest component of the psychiatry residency program. At the Department of Psychiatry and Mental Health at the Viseu Dão-Lafões Local Health Unit, residents are assigned to follow patients under the supervision of specialists and rotate through cases managed by different psychiatrists. This internship focuses on acute patients, with a predominant presence of affective and psychotic disorders.

**Objectives:**

This study aims to characterize patients hospitalized in an acute care unit, based on a sample monitored by the author.

**Methods:**

The data for this study was obtained from electronic health records systems used in Portugal, specifically Sclinico and Alert, covering information on patients I followed during the first nine months of 2024. Additionally, we conducted a literature review on this topic using PubMed.

**Results:**

The study sample comprised 20 female and 17 male patients. On average, the age of female patients is approximately 10 years higher than that of male patients, with women averaging 56 years and men averaging 46 years. The majority of male patients are hospitalized involuntarily under Portugal’s Mental Health Law, whereas this is less common among female patients.

The primary reason for hospitalization in men is psychotic decompensation within the context of schizophrenia, while affective disorders are predominant among female patients. The average length of stay is 45 days for male patients and 30 days for female patients.

**Conclusions:**

The longer average hospital stay for men may be linked to psychotic decompensation, often due to non-compliance with therapy. This lack of insight frequently results in involuntary hospitalization due to the risks posed to themselves and others. In contrast, affective disorders generally involve better-preserved insight, which could explain the shorter average hospital stays for women.

**Disclosure of Interest:**

None Declared

